# Internet Financial Data Security and Economic Risk Prevention for Android Application Privacy Leakage Detection

**DOI:** 10.1155/2022/6782281

**Published:** 2022-03-24

**Authors:** Yun Wang, Limei Wang

**Affiliations:** ^1^Sejong University, Seoul 05006, Republic of Korea; ^2^Xi'an University of Finance and Economics, Xi'an, Shaanxi 710100, China

## Abstract

The rapid development of the Internet has brought great convenience to our lives, but it has also brought many problems. Due to the virtual nature of the Internet, many criminals conduct illegal and criminal activities in the virtual world. In the Internet, ordinary users account for the vast majority of Internet users, but at the same time, the information of ordinary users is also the easiest to steal, and malicious behaviors of stealing information of ordinary users continue to occur. Android system and iOS system are the two most common systems in the current smart phone system market. In the face of the current Internet chaos, both systems have exposed problems to varying degrees, especially the Android system. In order to protect the privacy of users, researchers have also begun to focus on the privacy protection of the Android system. Today, with the rapid development of mobile payments, the privacy of mobile phones is closely integrated with the security of users' property, and the resolution of privacy issues cannot be delayed. Now that the development of the financial industry has developed into the Internet, the Internet has provided a new place for financial development, but it also faces many risks. This requires Internet finance practitioners to formulate corresponding security protection systems based on the characteristics of the Internet. Starting from big data and based on the characteristics of Internet finance, this paper designs a data-centric Internet financial risk early warning system. The existence of this system can analyze the possible risks of Internet finance from the perspective of big data, enabling enterprises to prepare in advance, and effectively reducing the losses in the development of Internet finance.

## 1. Introduction

Although the current Internet industry world has developed more maturely, the problem of privacy leakage still occurs from time to time [1]. In order to solve the problem of privacy leakage, researchers have developed more advanced analysis tools to detect whether there is privacy theft on the Internet [2]. However, when using these tools for detection, you need to rely on the dew point list to achieve, but it is very difficult to update and maintain the dew point list [3]. Especially in the Android system, a large amount of data are generated every time the list is updated, and the system cannot handle such a large amount of data, which makes the update of the list more difficult [4]. In order to improve this, this paper proposes a method of identifying privacy leaks based on machine learning. This method can provide researchers with richer information, while also speeding up system training and improving the recognition rate [5].

In addition, this article also proposes a method of detecting privacy leakage by detecting the path of privacy leakage [6]. This method is based on the small code as a starting point and based on its function diagram to detect whether there is illegal software in the Android system to steal privacy in this way, the theft can be monitored in real time [7]. In this article, a new algorithm and calculation model are creatively applied to process the data, which can reduce the complexity of calculation. In addition, we also designed a detection experiment. The experimental results show that the above method has excellent performance in detection efficiency and can improve the detection rate [8].

With the development of Internet technology, Internet finance has gradually emerged, and Internet finance has developed by relying on the Internet. The development of the Internet has provided a broad space for the development and promotion of the financial industry [9]. However, due to the various uncertainties in the Internet, Internet finance faces many risks in the development process [10]. In order to effectively avoid these risks, we should establish an early warning system for Internet finance [11]. The research focus of this article is the Internet financial risk early warning system with big data as the core. Through big data analysis, the possibility of risk occurrence and various risks that may be encountered during the development of Internet finance can be effectively calculated, so that enterprises can responding to risks in a timely manner and taking corresponding preventive measures have effectively promoted the healthy development of the Internet financial industry [12].

## 2. Related Works

Literature [13] mainly indicates the background and main purpose of the research of this article, and briefly describes the writing ideas and basic structure of this article. Literature [14] explains the reasons why Android system is prone to privacy leakage and how to detect the evil ways of privacy. First introduced the basic structure and operating mechanism of the Android system, and then conducted a research on the reverse analysis technology of Android, mainly clarifying the reverse analysis tool used by the Android system and how the tool plays a role in the Android system. After that, it introduces how the privacy leakage phenomenon of the Android system occurs and how to detect privacy leakage. Currently, the commonly used privacy leakage detection technologies are dynamic and static. Literature [15] first studied the LSSVM algorithm. Android APIs that have been classified by the LSSVM algorithm can be divided into three categories, and then they can be classified into 27 categories. Compared with other models, the detection model proposed in this paper has faster training speed and higher classification accuracy. Literature [16] first studied privacy leak detection technology in depth, and then proposed a better performance detection method based on the research results. This method is the Warshall algorithm, which can first compile information into small code, and then on this basis, a function call relationship graph related to it is constructed, and the algorithm can be optimized through the relationship graph, so as to obtain the accurate location of the privacy source and the privacy leakage point, and it can also detect the path of the privacy leakage, which can be more direct and accurate solve the problem of privacy leakage. This method is a static analysis method. Compared with the dynamic analysis method, the static analysis method is more accurate and easier to find the private path. It is a simple and easy to operate method. Literature [17] explains how to use applications to detect privacy leaks in the Android system. First, you need to design an application program that can detect privacy leaks and use static analysis and monitoring tool function module diagrams to explain the usage of the detection tool in detail through the program. Literature [18] is about how privacy leak detection is evaluated. First, experiments are needed to verify the accuracy of privacy leak detection. In the literature, relevant preparations for the test and the results and analysis of the experiment are accepted. According to the test results, we can analyze and evaluate different levels and types of leakage problems. Literature [19] discusses the design principles and design methods for the construction of Internet financial risk early warning systems from the perspective of big data. The Internet risk early warning system established from this perspective can enable enterprises to discover possible risks in the development process in a timely manner and improve their risk response capabilities.

## 3. Android Application Privacy Leakage Detection and Analysis Strategy

### 3.1. Privacy Source and Privacy Leakage Point Collection Technology Based on LSSVM Multi-Class Classification

#### 3.1.1. Theoretical Introduction

The concept of support vector machine was first proposed by a foreign scientist. This scientist gave the basic concept of support vector machine. With the continuous progress of science and the continuous progress of disciplinary research, the concept of support vector machine gradually improved. In processing data samples, it has the characteristics of strong classification ability and small number of samples, which effectively improves the calculation efficiency and is widely used in various fields. In addition, the support vector machine can also be applied to SVM, in which the original training set can be used for deep learning, and the sample data can be classified after learning.

In the application process, the situation of binary classification is first studied, and different classification situations are replaced by functions. The equation can be expressed by the following formula:

(1) $$\begin{array}{c} f\left(x\right)=\omega^{T}x+b=0. \end{array}$$

If it is classified in a multidimensional situation, two adjacent samples need to be separated in space. For a sample point to be classified, the sample should be separated from the plane as much as possible, as shown in the following formula:

(2) $$\begin{array}{c} \tilde{\gamma }=\gamma f\left(x\right)=y\gamma =\frac{\hat{y}}{\left\|\omega \right\|}. \end{array}$$

Therefore, the objective function of the maximum interval classifier can be defined as

(3) $$\begin{array}{c} \max\tilde{\gamma }\text{ s.t. }y_{i}=\left(\omega^{T}x_{i}+b\right)={\hat{\gamma }}_{i}\geq \hat{\gamma },\;i=1,2,\ldots ,n. \end{array}$$

The objective function is transformed into formula.

(4) $$\begin{array}{c} \max\frac{1}{\left\|\omega \right\|}=\min\frac{1}{2}{\left\|\omega \right\|}^{2},\\ \text{s.t},\;y_{i}\left(\omega^{T}x_{i}+b\right)\geq 1,\;i=1,2,\ldots ,n. \end{array}$$

When the penalty factor *C* and slack variables are introduced,

(5) $$\begin{array}{c} \min\frac{1}{2}{\left\|\omega \right\|}^{2}+C\sum_{i=1}^{n}\xi_{i},\\ \text{s.t. }y_{i}\left[\left(\omega \cdot x_{i}\right)+b\right]\geq 1-\xi_{i},\xi_{i}\geq 0,\;i=1,2,\ldots ,n. \end{array}$$

At the beginning, the image and space inside the circle could not be divided, so its features should be vectorized so that it can optimally segment the sample. But even this still has some problems, the most direct problem is the problem of computational complexity. To solve this problem, it is necessary to introduce the SVM kernel function, which can convert data into a high-latitude function through dot product operations. Therefore, when the sample cannot be classified, the kernel function can be introduced to transform it into a convenient separation form, and the optimal separation hyperplane can be constructed in a high-dimensional space.

(6) $$\begin{array}{c} f\left(x\right)=\mathrm{sgn}\left\{\sum_{i=1}^{n}a_{i}y_{i}K\left(x_{i},x\right)+b\right\}. \end{array}$$

#### 3.1.2. Collection Model of Privacy Sources and Privacy Leak Points

The values of all the extracted feature functions are one of the following two values: “1” means that the feature is applicable; “−1” means that the feature is not applicable, as shown in the following formula:

(7) $$\begin{array}{c} f_{i}=\left\{\begin{array}{cc} 1, & \text{featureapplication,}\\ -1, & \text{featuresnotapplicable,} \end{array}\right. \end{array}$$

where *f*_*i*_ represents the feature function, if the feature is applicable, the feature function value is 1, otherwise it is −1.

SVM is a secondary optimization problem. Its algorithm complexity is determined by the number of samples. The more samples, the more complex the SVM function algorithm. In this algorithm, there are constraints that can transform inequalities into equations. Conditional LSSVM, under this condition, the calculation of data can become simpler. For LSSVM, it is not only a constraint but also an algorithm, which is related to the complexity of the algorithm. The LSSVM classification problem can be described as

(8) $$\begin{array}{c} \min J\left(\omega ,b,e\right)=\frac{1}{2}{\left\|\omega \right\|}^{2}+\frac{1}{2}\gamma \sum_{i=1}^{n}e_{i}^{2},\\ \text{s.t. }y_{i}\left[\left(\omega \cdot x_{i}\right)+b\right]=1-e_{i},\;i=1,2,\ldots ,n. \end{array}$$

Among them, *γ* is the penalty factor, and ei is the slack variable. By defining the Lagrange function,

(9) $$\begin{array}{c} L\left(\omega ,b,e,a\right)=J\left(\omega ,b,e\right)-\sum_{i=1}^{n}a_{i}\left\{y_{i}\left[\left(\omega \cdot x_{i}\right)+b\right]-1+e_{i}\right\}, \end{array}$$

where *a*_*i*_ is the Lagrangian multiplier. According to the KKT condition, the formula (9) can be optimized to obtain

(10) $$\begin{array}{c} \left\{\begin{array}{c} \frac{\partial L}{\partial \omega }=0⟶\omega =\sum_{k=1}^{N}a_{k}y_{k}\phi \left(x_{k}\right),\\ \frac{\partial L}{\partial b}=0⟶\sum_{k=1}^{N}a_{k}y_{k},\\ \frac{\partial L}{\partial e_{k}}=0⟶a_{k}=\gamma e_{k},\\ \frac{\partial L}{\partial a_{k}}=0⟶y_{k}\left[\omega^{T}\phi \left(x_{k}\right)+b\right]-1+e_{k}=0. \end{array}\right. \end{array}$$

The solution process of the above equation can be converted to the linear equation system

(11) $$\begin{array}{c} \left(\begin{array}{cc} \text{0} & -Y^{T}\\ Y & ZZ^{T}+\gamma^{-1}I \end{array}\right)\cdot \left(\begin{array}{c} b\\ a \end{array}\right)=\left(\begin{array}{c} 0\\ 1 \end{array}\right). \end{array}$$

Among them, *Z* = (*φ* (*x*_1_)^*T*^*y*_1_,…, *φ* (*x*_N_)^*T*^*y*_*N*_); *Y* = (*y*_1_, *y*_2,_ …, *y*_*N*_): *e* = (*e*_1_, *e*_2_,…, *e*_*N*_); *I* is Identity matrix; *a* = (*a*_1_, *a*_2_,…, *a*_*N*_). Then the decision function of LSSVM is

(12) $$\begin{array}{c} f\left(x\right)=\mathrm{sgn}\left\{\sum_{i=1}^{n}a_{i}y_{i}K\left(x_{i},x\right)+b\right\}. \end{array}$$

The method of constructing one-to-one two-class classifiers to perform DAG operations is also called the DAGSVM method. There are several methods similar to this method, but the other methods cannot meet the needs of this article, only this the method is most suitable for the research of this article. Therefore, the other two methods will not be described in detail in this article.

The following compares the one-to-many and one-to-one algorithms, and the training learning time is shown in the following equation.

(13) $$\begin{array}{c} T=cm^{\gamma }. \end{array}$$

It can be seen from the above formula that the learning and training time of SVM is directly determined by the complexity of the algorithm, and the training time of the SVM multiclass classifier indirectly depends on the classification rate of the classifier. In the one-to-many algorithm, due to the construction of multiple functions, the training of these functions will consume a lot of time, so it can be concluded that the training time is

(14) $$\begin{array}{c} T=kcm^{\gamma }. \end{array}$$

However, only one is constructed in the one-to-one algorithm, so the training time can be obtained as shown in formula.

(15) $$\begin{array}{c} T=\frac{k\left(k-1\right)}{2}C\left(\frac{2m}{k}\right)\approx 2^{\gamma -1}k^{2-\gamma }cm^{\gamma }. \end{array}$$

#### 3.1.3. Experimental Results and Analysis

Common kernel functions can map input variables to high-dimensional space. RBF cannot show good performance due to insufficient empirical information among these kernel functions. Moreover, RBF has its own shape parameters, so there are fewer calculation steps and simple calculations. This article uses the reason for the RBF model. The kernel function selected in this paper is compared with other kernel functions as shown in Table 1.

It can be seen from Table 1 that the accuracy of the RBF kernel function is higher than the other two functions, so we use the RBF function for data classification, and Table 2results after classification are shown in the following table.

For the second classification, that is, the breakdown of privacy sources and privacy leaks, and the results are shown in Table 3.

It can be seen from Table 3 that the accuracy of the model test is not high. This is because the API training data are too small, and there is not enough data for classification.

### 3.2. Theory Introduction

In the above, although we defined the privacy source and the detection of privacy leakage, we did not give the definition of the privacy leakage path. We will give the relevant definitions below.

**Definition 1 .**

The path of privacy leakage: that is, how privacy is leaked and the trend after it is leaked.

Privacy leakage path detection is mainly to detect the privacy leakage path in the application software, but there will be various paths in the system. How to detect the leakage path in the shortest time is the problem that this method needs to solve.

**Definition 2 .**

Let *G* = (*V*, *E*) be a directed graph of order *n*, the other *V* represents a collection of points, and *E* represents a collection of edges.

**Definition 3 .**

The adjacency matrix of the directed graph *G* = (*V*, *E*) is *W* = (*W*_*ij*_)_*n*×*n*_, as shown in the following formula:

(16) $$\begin{array}{c} W_{ij}=\left\{\begin{array}{cc} 1, & M_{\left(i\right)}\text{ to }M_{j}\text{ havenon}-\text{zeroroutes,}\\ \text{0,} & \text{other.} \end{array}\right. \end{array}$$

In order to detect the privacy leak path as quickly as possible, we use the Warshall algorithm to detect it. According to the functional relationship, it is transformed into a directed graph to calculate its reachability. The final matrix is as

(17) $$\begin{array}{c} R_{ij}=\left\{\begin{array}{cc} 1, & M_{\left(i\right)}\text{ to }M\left(j\right)\text{havenon}-\text{zeroroutes,}\\ \text{0,} & \text{other.} \end{array}\right. \end{array}$$

#### 3.2.1. Component-Level Privacy Leakage Detection Model Construction

The Android system is composed of a variety of components, and component development can be used to make the code structure clearer and improve compilation efficiency. Therefore, when analyzing the problem of privacy leakage, it is necessary to analyze from the components. By analyzing the data of the model, the data of the system can be quickly obtained to improve operating efficiency. In the detection process, it is necessary to detect the stains between the components, which can ensure a more comprehensive and thorough detection of the privacy leakage path.

We can build a model according to the specific process of privacy leak detection, and the general idea is as follows:

1. Derive the system from the result to get the corresponding byte and code;
2. Drawing according to bytes and codes;
3. Drawing using Soot's plug-in;
4. Combine the figures drawn in (2) and (3) to form a new figure;
5. Perform stain detection on components;
6. The test result is manually tested by the tester.

**Figure 1:**
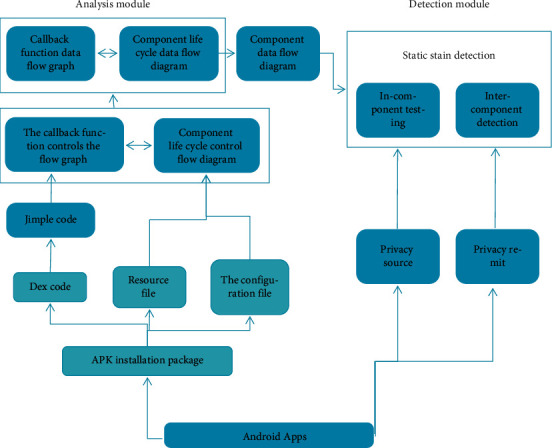
Model diagram of privacy leak detection mechanism.

According to the above steps, the specific detection mechanism model diagram is shown in Figure 1.

CFG is composed of a set of nodes and connecting nodes. The successor set of nodes can be expressed as

(18) $$\begin{array}{c} \text{Before}\left(b\right)=\left\{n\in N|\exists e\in E,\;e=b-n\right\}. \end{array}$$

The set of nodes that can be derived from the confluence node can be expressed as

(19) $$\begin{array}{c} \text{After}\left(b\right)=\left\{n\in N|\exists e\in E,\;e=n-b\right\}. \end{array}$$

For a basic block, there are four corresponding sets. The four sets have different meanings. The correspondence between the four sets is as follows:

(20) $$\begin{array}{c} \text{output}\left(a\right)=\left(\text{input}\left(a\right)-\text{remove}\left(a\right)\right)\cup \text{create}\left(a\right),\\ \text{input}\left(a\right)=U\left(\text{output}\left(b\right)\right). \end{array}$$

Before the data flow graph is generated, it is necessary to analyze the input value and output value of each basic block in the application.

#### 3.2.2. Optimization and Improvement of Warshall Algorithm

It can be seen from the above algorithm that the time complexity of this algorithm is *O* (*n*_3_). In this process, we used the Warshall algorithm to construct the transitive closure relationship based on the matrix. Among them, this series of *n*-order matrices is shown in the following equation (21). Assume

(21) $$\begin{array}{c} R^{\left(0\right)}\text{,}R^{\left(1\right)},R^{\left(2\right)}\text{,}\ldots \text{, }R^{\left(n\right)}. \end{array}$$

Each *R*^(*i*)^ matrix contains a directed graph, so the core formula of Warshall algorithm can be derived:

(22) $$\begin{array}{c} R^{\left(k\right)}\left[i,j\right]=R^{\left(k-1\right)}\left[i,j\right]\text{or}\left(R^{k-1}\left[i,k\right]\&\&R^{\left(k-1\right)}\left[k,j\right]\right). \end{array}$$

**Figure 2:**
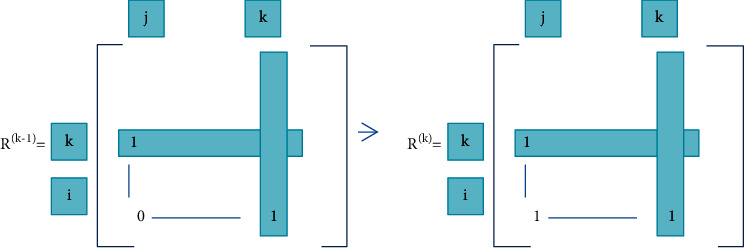
Warshall algorithm set 1 rule.

The above formula (22) can be divided into the following two situations according to the different values of *R* [*i*, *j*] in *R*^(*k*−1)^:

1. If a node *R* [*i*, *j*] is 1 in *R*^(*k*−1)^, it is still 1 in *R* (*k*);
2. If a node *R* [*i*, *j*] is 0 in *R*^(*k*−1)^ if and only if it is in *R* (*k* − 1), the *i*th row and *k*th column, *k*th row and *j*th column The nodes of are all 1, which can be set to 1 in *R* (*k*), as shown in Figure 2.

**Figure 3:**
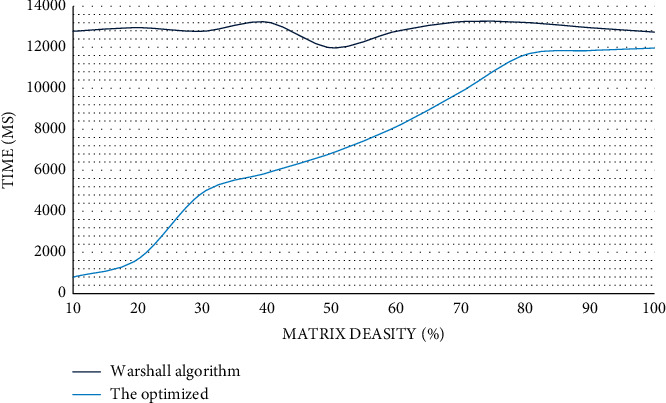
Comparison of time complexity before and after Warshall algorithm optimization.

Since in the function call relationship graph, Android applications are basically not used in the case of pairwise correspondence; therefore, most of the matrix models of the call relationship graph are sparse models. It can be seen from Figure 3 that when *R*^(*k*−1)^ [*k*, *j*] is 1, the value of *R*^(*k*)^ [*i*, *j*] has the following three situations:

(23) $$\begin{array}{c} \text{when }R^{k-1}\left[i,j\right]=1,R^{\left(k\right)}\left[i,j\right]=1, \end{array}$$

(24) $$\begin{array}{c} \text{when }R^{\left(k-1\right)}\left[i,j\right]=0,R^{\left(k-1\right)}\left[i,k\right]=1,R^{\left(k\right)}\left[i,j\right]=1, \end{array}$$

(25) $$\begin{array}{c} \text{when }R^{\left(k-1\right)}\left[i,j\right]=0,\text{if }R^{\left(k-1\right)}\left[i,k\right]=0,R^{\left(k\right)}\left[i,j\right]=0. \end{array}$$

Among them, formula (23) can be equivalent to the following form:

(26) $$\begin{array}{c} \text{when }R^{\left(k-1\right)}\left[i,j\right]=1,R^{\left(k\right)}\left[i,k\right]0=R^{\left(k-1\right)}\left[i,j\right]. \end{array}$$

Formula (24) and formula (25) can be equivalent as follows:

(27) $$\begin{array}{c} \text{when }R^{\left(k-1\right)}\left[i,j\right]=0,R^{\left(k\right)}\left[i,j\right]=R^{\left(k-1\right)}\left[i,k\right]. \end{array}$$

Combining formula (26) and formula (27) is obtained:

(28) $$\begin{array}{c} \text{when }R^{\left(k-1\right)}\left[k,j\right]=1,R^{\left(k\right)}\left[i,j\right]=R^{\left(k\right)}\left[i,j\right]\vee R^{\left(k-1\right)}\left[i,k\right]. \end{array}$$

#### 3.2.3. Experimental Results and Analysis

**Figure 4:**
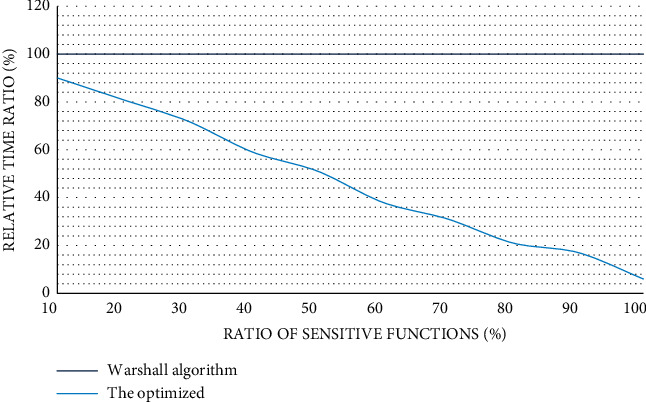
The influence of the proportion of sensitive functions on the detection algorithm.

(1) For the optimization of Warshall algorithm, the results obtained through experiments are shown in Figure 4 below.

**Figure 5:**
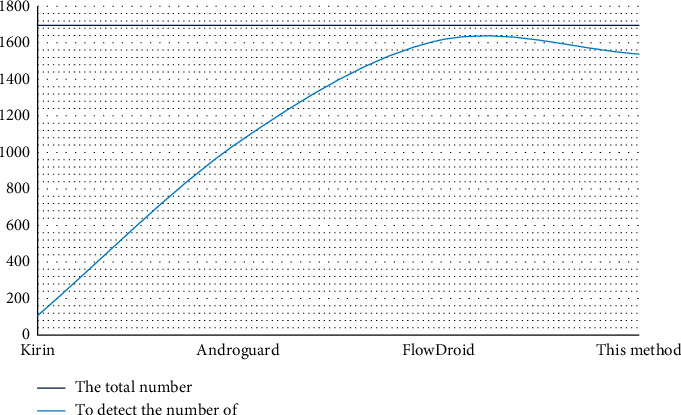
Availability detection.

It can be seen from Figure 3 that the relationship between the values represented by the horizontal axis and the vertical axis is proportional. And, in Android applications, most of the function models are sparse models, which illustrates the superiority of the algorithm to a certain extent. We obtained the data shown in Figure 5 through experiments:

It can be seen from Figure 4 that the more sensitive functions account for, the less the relative time ratio is. From this, we can also get a detection algorithm that improves efficiency.

(2) The test results in this article are compared with the situation in real life, and the indicators in Table 4 are obtained.

Recall rate = actual number detected/total number to be detected.

False alarm rate = number of false detections/total number of detections.

Correct rate = number of correct detections/total number of detections.

The test results are shown in the table below.

(3) For usability detection, we compared Kirin, Androgurad, and FlowDroid and got the data in Figure 5:

The calculation process of Androguard is different from other methods. This method can often only detect malicious programs with known characteristics. However, in actual situations, most malicious programs do not know the characteristics, and new malicious programs are constantly being generated. We cannot get their characteristics one by one, which is time-consuming and laborious, and does not meet the needs of this article. The method needed in this article is a method that can accurately and effectively detect privacy leaks and eliminate taints between components in the system.

### 3.3. Implementation of Android Application Privacy Leakage Detection Tool

**Figure 6:**
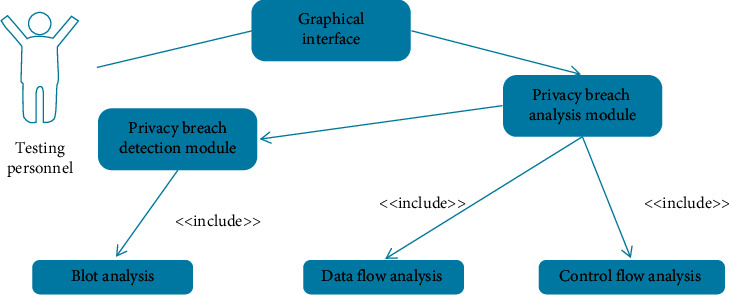
Test tool use case diagram.

**Figure 7:**
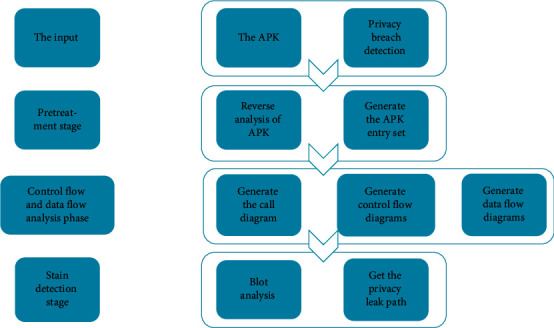
Privacy leak detection mechanism.

The analysis of the Android application privacy leak detection process is shown in Figure 6:

CoDroid's detection of Android application privacy leakage is mainly divided into three stages, as shown in Figure 7.

### 3.4. Experimental Design and Result Analysis

#### 3.4.1. Experimental Environment Design

The experimental environment is shown in Table 5:

The Source and Sink lists used in this project are calculated using a foreign university.

Table 6 shows the privacy data sources related to location information; Table 7 shows the privacy data collection related to calendars.

In the development process of Android applications, the traces and information of the development process are often recorded in the log for the convenience of the next use. This makes the log contain a lot of private data, and the private information in the log enables the user to more convenient and faster use of the application, but if you do not pay attention to the encryption of the log, it is likely to pose a threat to the user's privacy and even the security of the property.

#### 3.4.2. Privacy Leak Detection and Effectiveness

This experiment randomly selected two hundred samples from the major Android application markets. The two hundred samples were all randomly selected qualified samples on the market, and the utilization rate of these applications and the proportion of users were very large. Through the analysis of these samples, it is found that more than a quarter of the samples have leaked user privacy behavior. The specific data are shown in Table 8 below:

Through analyzing the process of privacy leakage, we found that different privacy data leakages are mainly:

1. Equipment-related information.
2. Call history and application chat history.
3. Position positioning.
4. Information sent/received.

The statistical results are shown in Table 9:

## 4. Internet Financial Data Security Analysis and Economic Risk Prevention Strategies

### 4.1. Analysis of Data and Its Characteristics in Internet Finance

Internet financial data have the characteristics of large scale, wide variety, and fast update speed. Large-scale Internet finance has a large amount of data, and with the continuous development of the Internet finance industry, the amount of data is still increasing. On the other hand, as a low-threshold, low-cost platform, the Internet has relatively low requirements for users, which has led to uneven quality of Internet users. Now, more and more people have poured into the Internet finance industry and injected them the vitality also brings many problems.

The variety refers to the types of Internet data, and it is also reflected in the wide variety of services provided by Internet finance.

Fast update speed is a necessary requirement for the Internet finance industry to ensure advancement and real-time. The Internet financial industry has very high requirements for data, requiring accurate data while ensuring real-time data.

In addition, the risk early warning system of Internet finance is also based on data. Therefore, to realize the timely monitoring and management of Internet finance, it is necessary to fully understand the three characteristics of Internet finance data and make full use of these three characteristics.

### 4.2. Categories of Economic Crime Risks in the Internet Financial Environment

The characteristics of the Internet determine that the Internet is difficult to manage, so there are more and more financial crimes on the Internet platform. The main types of crimes are as follows.

#### 4.2.1. Making Loans on the Internet

The risk of making loans on the Internet is high, and most platforms do not pay attention to the protection of user privacy. There are even platforms that illegally sell user information. This financial management model is also easily used by criminals to commit economic crimes.

#### 4.2.2. Third-Party Payment Platform Risks

With the popularization of mobile payment, third-party payment has become an indispensable payment tool in people's daily life and even replaced advanced ones. However, there are also many risks in the third-party payment process. Criminals will steal users from third parties. The privacy information of the payment platform causes users to suffer property or privacy losses.

#### 4.2.3. Risk of Illegal Fund-Raising Crime

Perpetrators of this type of criminal use online platforms to establish false online lending platforms and use money as bait to lure the public who do not know the truth to invest funds. After the public invests a certain amount of money, the perpetrators of the platform will run away with money. Money is generally difficult to recover. In the past two years, such cases have occurred more and more frequently, causing losses to many Internet users.

#### 4.2.4. Risk of Money Laundering Crime

Online transactions are very convenient. People can buy their favorite items home with a simple movement of their fingers. This not only facilitates people's lives but also provides “convenience” for criminals' money laundering behavior. This type of money laundering crime is generally difficult to find key evidence, which makes criminals more arrogant.

### 4.3. Preventive Measures against Economic Crime Risks in the Internet Financial Environment

#### 4.3.1. Learn from Foreign Advanced Legislation Experience and Promote the Diversification of Online Legislation

With the continuous promotion of Internet applications, the phenomenon of Internet financial crimes is becoming more and more common. Internet financial crimes are often difficult to capture and severely endanger social order. Currently, China has insufficient experience in dealing with Internet financial crimes. It is necessary to actively learn from advanced foreign countries.

#### 4.3.2. Strengthen International Judicial Cooperation

Internet financial crimes are generally international. Criminals flee the world's networks and are difficult to be caught. To solve Internet financial problems requires the power of the international community, and the whole world must unite and cooperate to combat Internet financial crimes.

#### 4.3.3. Strengthen Diversified Supervision

Generally speaking, more developed countries pay more attention to the management of online finance. Western countries headed by Country M signed relevant laws and regulations as early as 2010.

#### 4.3.4. Improve the Quality of Staff in Relevant Departments

Criminals in online finance are often people with high IQ who have a certain economic foundation and antireconnaissance capabilities, which also makes it difficult to detect online financial crimes. This requires cyber police to have a high level of professionalism. So far, the public security department has set up a dedicated cyber surveillance bureau, but China's cyber surveillance agency is still not sound, especially in terms of technical investigations. Technical investigation capabilities and talents that meet the needs. In addition, the lack of close contact between network operators and judicial departments also makes it difficult to carry out network investigations, and criminals cannot be caught in time. In order to solve the above-mentioned problems, the government must play a leading role, closely link relevant departments, and formulate corresponding policies to effectively improve the monitoring, early warning, and work capabilities of relevant departments.

## 5. Conclusion

Today, smart mobile terminals are used more and more frequently, whether in communications, entertainment, or shopping, they are inseparable from smart mobile terminals. People's dependence on mobile devices makes mobile devices more and more stored user's private information. This makes the mobile terminal not only bring convenience to people but also bring certain risks to people. The security maintenance of privacy has become a key factor that threatens user privacy. Aiming at the hot issues of Android system application privacy leakage detection, this topic uses various analysis techniques and models to monitor applications and develops an algorithm tool specifically for the Android system according to the characteristics of the Android system. The tool can effectively monitor the applications in the Android system and closely monitor privacy leaks. In order to solve the problem of privacy leakage, researchers use sophisticated technical tools to detect users' private data and determine whether there is a privacy leakage problem. However, most of the current privacy detection tools rely on lists for detection. This detection method has certain limitations, and with the update of the Android system, new privacy leaks will continue to be generated. This will seriously lead to the accuracy of privacy leak detection, and at the same time cannot guarantee the privacy of users. In order to improve this, this paper proposes a privacy detection method that uses machine learning instead of traditional methods. With the rapid development of the Internet, crimes in the virtual world have become more and more rampant. Internet financial crimes have become the most common type of financial crimes. This is because the crime costs of Internet financial crimes are low, and the behaviors are relatively low. Concealed, Internet financial crimes have seriously endangered social order. Relevant departments should actively respond to Internet financial crimes and privacy leakage issues and formulate corresponding laws and regulations to prevent illegal crimes. This article mainly studies the Internet financial data security and economic risk prevention for Android application privacy leakage monitoring, but there are still some shortcomings in the classification of privacy sources. In the future, we will optimize and improve these shortcomings so that it can more accurately and effectively monitor privacy leaks.

## Figures and Tables

**Table 1:** Comparison of accuracy rates of three kernel functions.

Kernel function type	Linear kernel (%)	Polynomial kernel function (%)	RBF kernel function (%)
Accuracy	92.3	84.62	92.5

**Table 2:** Model test results.

Classification	Recall rate (%)	Accuracy (%)
Privacy source	92.9	89.2
Privacy leaks	87.4	89.6
Other API	94.9	95.1
Weight average	93.0	93.1

**Table 3:** Model test results.

Classification	Recall rate (%)	Accuracy (%)
Privacy source classification (average weight value)	88.5	89.9
Classification of privacy leak points (average weight)	86.4	88.7

**Table 4:** Correctness test results.

Total number of detection applications	697
Check the full quantity	681
Recall rate	97.7%
Number of false positives	25
False alarm rate	3.59%
Correct rate	94.12%

**Table 5:** Experimental environment.

Project	Configuration value
Operating system	Window7 64 bit
CPU	Inter(R) Core(TM)i3-2350M CPU@2.30 GHz
RAM	8.00 G
Java runtime environment variables	JAVA_HOME: C:\Program Files\Java
SDK version	Android-17, android-18, android-19, android-20
	D:\Soot\adt-bundle-windows-x86_64-20131030\
SDK path	Adt-bundle-windows-x86_64-20131030\sdk\platforms

**Table 6:** Privacy data sources for location information.

API function	API
Get latitude information	<android.location.Location: double get [attitude(>
Get longitude information	<android.location.Location: double getLongitude()>
	<android.location.LocationManager:getIastKnownLocation
GPS positioning to obtain location information	(java.lang.String)>
	getAddress()>
Bluetooth positioning to obtain location information	<android.bluetooth.BluetoothAdapter: java.lang. String
WiFi location to obtain MAC address	<android.nt.wifi.WifiInfo:java.lang.String:getMac Address()>
WiFi positioning to obtain SSID information	<android.net.wifi.WifiInfo:java.lang.StringgetSSID()>
Base station location to obtain CID	<android.telephony.gsm.GsmCelIL ocation: int getCid(>
Base station positioning to obtain location	<android.telephony.gsm.GsmCelLocation:intgetLac(>

**Table 7:** Summary of privacy data about logs.

API function	API
Debug type log output	<android.util.Log: int
	djava.lang. String.java.lang.String)>

Error type log output	<android.util.Log: int
	e(java.lang.String.java.lang.String)>

Log output of prompt message type	<android.util.Log: int
	i(java.lang.String, java.lang.String)>

Warning type log output	<android.util.Log: int
	w(java.lang.Stringjava.lang.String)>

All types of log output	<android.util.Log: int
	v(java.lang.Stringjava.lang.String)>

While outputting the log, print out the execution path (call stack) of the code at this time	<android.util.Log: int
	wtf(java.lang.String, java. lang.String)>

**Table 8:** Statistics of the number of programs that leaked user privacy information.

Types of leaked private data	Number of programs (a)	Percentage (%)
Equipment related information IMEI	45	59.2
Contacts and call records	16	21.1
Location information	10	13.1
Short message	4	5.3
Log information	1	1.3

**Table 9:** Statistics of privacy breach destinations.

Types of leaked private data	Total number of leaks (a)	Leaked to the developer (a)	Leaked to advertisers (number)
Equipment-related information IMEI	45	32	13
Contacts and call records	16	11	5
Location information	10	4	2
Short message	4	4	0
Log information	1	1	0

## Data Availability

The data used to support the findings of this study are available from the corresponding author upon request.

## References

[1] Cheng Y. Y., Ying L. Y. B., Jiao S. B. R., Su P. R. G., Feng D. G. (2014). Research on user privacy leakage in mobile social messaging applications. *Jisuanji Xuebao/Chinese Journal of Computers*.

[2] Wang K. (2012). *Research and Application of Android Platform Application Risk Detection*.

[3] Li T. X., Xing Y. X. Q., Hu A. Q. J., Wang Y. J. (2018). Research on multi-dimensional privacy leakage evaluation model for mobile terminals. *Jisuanji Xuebao/Chinese Journal of Computers*.

[4] Van Schaik P., Jansen J., Onibokun J., Camp J., Camp J., Kusev P. (2018). Security and privacy in online social networking: risk perceptions and precautionary behaviour. *Computers in Human Behavior*.

[5] Dini G., Martinelli F., Matteucci I., Petrocchi M., Saracino A., Sgandurra D. (2018). Risk analysis of Android applications: a user-centric solution. *Future Generation Computer Systems*.

[6] Chen H., Ünal A. B., Akgün M., Pfeifer N. Privacy-preserving SVM on outsourced genomic data via secure multi-party computation.

[7] Huo Z. F., Meng X. F., Huang Y. (2013). PrivateCheckIn: trajectory privacy-preserving for check-in services in MSNS. *Jisuanji Xuebao/Chinese Journal of Computers*.

[8] Zhang Y. Q. Q., Lv S. Q., Fan D. (2015). Anomaly detection in online social networks. *Jisuanji Xuebao/Chinese Journal of Computers*.

[9] Hasan M. M., Popp J., Oláh J. (2020). Current landscape and influence of big data on finance. *Journal of Big Data*.

[10] Ma X., Lv S. (2019). Financial credit risk prediction in Internet finance driven by machine learning. *Neural Computing & Applications*.

[11] Ma L., Wang Y., Ren C., Li H., Li Y. (2020). Early warning for internet finance industry risk: an empirical investigation of the P2P companies in the coastal regions of China. *Journal of Coastal Research*.

[12] Lin Z., Whinston A. B., Fan S. (2015). Harnessing Internet finance with innovative cyber credit management. *Financial Innovation*.

[13] Shang M., Chen M. (2014). A calculation method of risk value of information assets. *Network security technology and application*.

[14] Chen L., Lu R., Alharbi K., Lin X., Cao Z. (2015). ReDD: recommendation-based data dissemination in privacy-preserving mobile social networks. *Security and Communication Networks*.

[15] Liang J., Qin Z., Ni J., Lin X., Shen X. Efficient and privacy-preserving outsourced SVM classification in public cloud.

[16] Ziegeldorf J. H., Morchon O. G., Wehrle K. (2014). Privacy in the Internet of Things: threats and challenges. *Security and Communication Networks*.

[17] Li H., Zhu H., Du S., Liang X., Shen X. (2016). Privacy leakage of location sharing in mobile social networks: attacks and defense. *IEEE Transactions on Dependable and Secure Computing*.

[18] Han G., Shu L., Chan S., Hu J. (2016). Security and privacy in Internet of Things: methods, architectures, and solutions. *Security and Communication Networks*.

[19] Gu G., Zhu W. (2021). Time-varying transmission effects of Internet finance under economic policy uncertainty and internet consumers behaviors: evidence from China. *Journal of Advanced Computational Intelligence and Intelligent Informatics*.

